# A Case Series of Multiple Primary Malignancies Among Patients With Advanced Melanoma

**DOI:** 10.7759/cureus.15480

**Published:** 2021-06-06

**Authors:** Matthew I Ebia, Stephen Capone, Charité Ricker, Jacob S Thomas, Varsha Tulpule, Irene Kang, Anishka D'Souza, David R Freyer, Kimberly Miller, Gino K In

**Affiliations:** 1 Department of Internal Medicine, Los Angeles County University of Southern California Medical Center, Los Angeles, USA; 2 Department of Neurosurgery, MD Anderson Cancer Center, Houston, USA; 3 Division of Oncology, University of Southern California - Keck School of Medicine, Norris Comprehensive Cancer Center, Los Angeles, USA; 4 Department of Preventive Medicine, University of Southern California - Keck School of Medicine, Los Angeles, USA; 5 Departments of Preventive Medicine and Dermatology, University of Southern California - Keck School of Medicine, Los Angeles, USA; 6 Department of Dermatology, University of Southern California - Keck School of Medicine, Los Angeles, USA

**Keywords:** multiple primary malignancies, melanoma, immunotherapy, hereditary cancer, cancer survival

## Abstract

Multiple primary malignancies (MPM) are described as two or more primary tumors within the same individual. The impact of MPM on the tumor microenvironment among patients with melanoma is poorly understood. Here, we describe this unique group of patients who have both advanced melanoma and at least one other primary malignancy and report their survival outcomes. In this study, patients with advanced melanoma and a second primary malignancy were identified. Medical records were reviewed for cancer treatment history. Kaplan-Meier methods were used to derive survival curves and estimate overall survival (OS), and log-rank tests were used to compare OS. Among 11 MPM patients, the most common non-melanoma cancers were breast (n = 3) and thyroid (n = 3). Median OS was 153.5 months for all patients. Median OS for synchronous MPM (sMPM) and metachronous MPM (mMPM) were 83.1 and 196.7 months, respectively (p= 0.10). Median OS was not reached when melanoma was diagnosed first, and 153.5 months when diagnosed second (p= 0.45). For six patients receiving immunotherapy for melanoma, there was a 100% complete response rate. In conclusion, patients with melanoma are at risk of secondary malignancies, including breast and thyroid cancer. The timing of secondary malignancies may impact prognosis. Further study of the impact of immunotherapy on MPM is warranted.

## Introduction

Multiple primary malignancies (MPM) describe two or more primary tumors arising in the same individual [1-3]. The reported frequency of MPM is between 2% and 17% of all cancers [2,4]. Risk factors for MPM include hereditary syndromes, environmental exposures, hormonal factors, immune deficiency, infection, carcinogenic effects of prior cancer therapies, and/or a combination of these [4]. The development of more than two MPM is increasingly rare with only 1.4% of patients diagnosed with three malignancies and 0.2% with four or more [5].

Malignancies which derive from a shared etiological factor (e.g., tobacco, alcohol, or infection) may co-occur in the same individual due to field cancerization. For example, the co-occurrence of lung cancer and head/neck cancer is a well-described pattern among patients with a history of smoking. However, for cancers that do not have a common carcinogenic exposure, patterns of MPM incidence are less clear. In addition, prior cancer therapies, such as radiotherapy or chemotherapy, may also lead to development of a second cancer.

Melanoma is an aggressive skin cancer with the potential for local invasion and metastasis to other parts of the body. Although UV radiation is the primary etiology for cutaneous melanoma, patients with melanoma are at risk of developing MPM such as breast, prostate, colorectal, kidney, and non-Hodgkin lymphoma, in addition to a second primary melanoma or other skin cancers [5,6].

While it is clear that melanoma may cause immune suppression at the individual level, the molecular underpinnings of immune suppression among patients with MPM, where multiple tumors may simultaneously be exerting suppressive effects, remain to be elucidated. Furthermore, the role of immune suppression is of particular concern for patients with advanced melanoma, where regional or distant metastatic disease leads to poor outcomes. Here, we attempt to describe this unique group of patients who have both advanced melanoma and at least one other primary malignancy.

## Materials and methods

In this retrospective study, patients with advanced melanoma and at least one other non-melanoma malignancy (excluding non-melanoma skin and in situ cancers) were identified from a clinical cancer program registry between 2012 and 2020. Advanced melanoma was defined as stage III-IV disease by American Joint Committee on Cancer (AJCC) 8th edition staging. Patient demographics and cancer history were recorded. Synchronous cancers (sMPM) were defined as those occurring within six months of the first primary malignancy whereas metachronous cancers (mMPM) were defined as those occurring at least six months after the first primary. We included both patients who had melanoma first before a second malignancy (MEL1), and those who had a non-melanoma malignancy first, followed by melanoma later (MEL2). Patients with more than one primary melanoma were excluded. Overall survival (OS) was measured as time from diagnosis of the first cancer to death due to any cause; subjects who were alive and lost to follow-up at the time of last encounter were censored. Log-rank tests and chi-squared values were used to compare the OS among these groups with statistical significance defined as p value < 0.05. Kaplan-Meier methods were used to derive survival curves and estimate OS. This study was approved by the Institutional Review Board of the University of Southern California.

## Results

An initial cohort of 15 patients with melanoma and MPM was identified; four patients were excluded based on the absence of advanced melanoma (stage II or lower). Among 11 patients included for analysis (Table 1), the median age was 59 years (range: 45-75); 64% were male, and 36% female, with 73% White/Caucasian, and 27% Hispanic. Median duration of follow-up was 64.9 months (range: 8-240 months). The majority had stage III melanoma (72.7%, n = 8), while 27.3% (n = 3) had stage IV. Among nine patients with somatic mutation testing performed on melanoma tumors, there were four NRAS Q61R mutations, three BRAF V600E mutations, and one loss-of-function NF1 mutation. Melanoma treatment included surgery (90.9%, n = 10), radiation (9.1%, n = 1), and systemic therapy (e.g., cytotoxic chemotherapy, cytokine-based immunotherapy, or targeted) (81.8%, n = 9), with 54.5% (n = 6) receiving immune checkpoint inhibitors (ICI) (e.g., PD-1 and/or CTLA-4 inhibitor).

**Table 1:** Demographic and tumor-related variables for all patients with advanced melanoma and second primary malignancy

Patient	Age	Sex	Ethnicity	First Cancer	Stage	Treatment	Second Cancer	Stage	Treatment	Third Cancer	Stage	Treatment	Fourth Cancer	Stage	Treatment
1	57	F	Caucasian	Breast	IIIA T2N2MX	Surgery, chemotherapy, hormone therapy	Melanoma (upper extremity)	IIIB T1N1MX	Surgery, interferon						
2	61	F	Caucasian	Melanoma (lower extremity)	IIIB T2N1MX	Surgery, PD-1 inhibitor	Breast	IIB TXN1MX	Surgery, chemotherapy, hormone therapy						
3	57	M	Caucasian	Colorectal	IV T3N1M1	Surgery, chemotherapy	Melanoma (head/neck)	IV T1NXM1	Surgery, PD-1 inhibitor						
4	75	M	Caucasian	Melanoma (head/neck)	IIIB T2N1MX	Surgery, PD-1 inhibitor	Colorectal	IIIB T4N1MX	Surgery, chemotherapy						
5	58	M	Caucasian	Pancreas	IV TXNXM1	Surgery	Melanoma (unknown primary)	III TxN1MX	None						
6	62	M	Caucasian	Melanoma (unknown primary)	IIIB TXN1MX	Surgery, CTLA4 + PD-1 inhibitor	Renal clear cell carcinoma	I T1NXMX	Surgery						
7	59	F	Hispanic	Melanoma (upper extremity)	IIIC T4N1MX	Surgery, radiation, interferon, targeted therapy	Renal clear cell carcinoma	IV T3NXM1	Surgery, chemotherapy, radiation						
8	45	M	Hispanic	Melanoma (lower extremity)	IIIC T3N2MX	Surgery, PD-1 inhibitor	Papillary thyroid carcinoma	I T1NXMX	Pending surgery						
9	58	M	Caucasian	Melanoma (upper extremity)	IIIB T3N1MX	Surgery, interferon	Papillary thyroid carcinoma	III T1N1MX	Surgery						
10	66	F	Caucasian	Breast	IA T1N0MX	Surgery, hormone therapy, chemotherapy	Melanoma (upper extremity)	IIIC T3N2MX	Surgery, CTLA-4 inhibitor, radiation, PD-1 inhibitor	Chronic myeloid leukemia	N/A	Oral TKI therapy			
11	73	M	Hispanic	Prostate	Unknown	Surgery	Lung	IIIA T2N2MX	Chemoradiation, PD-1 inhibitor, chemotherapy	Melanoma (head/neck)	IIIC T4N1MX	Surgery	Papillary thyroid carcinoma	I T1NXMX	Surgery

Among the 11 patients, there were five sMPM, and six mMPM. Six patients presented with MEL1 and five with MEL2. The median time between diagnosis of first primary to second primary cancer was 7.1 months. For sMPM and mMPM, the median time between diagnoses was 1.8 and 19.3 months, respectively. For MEL1 and MEL2, the median time between diagnoses was 13.6 and 4.5 months, respectively.

**Figure 1:**
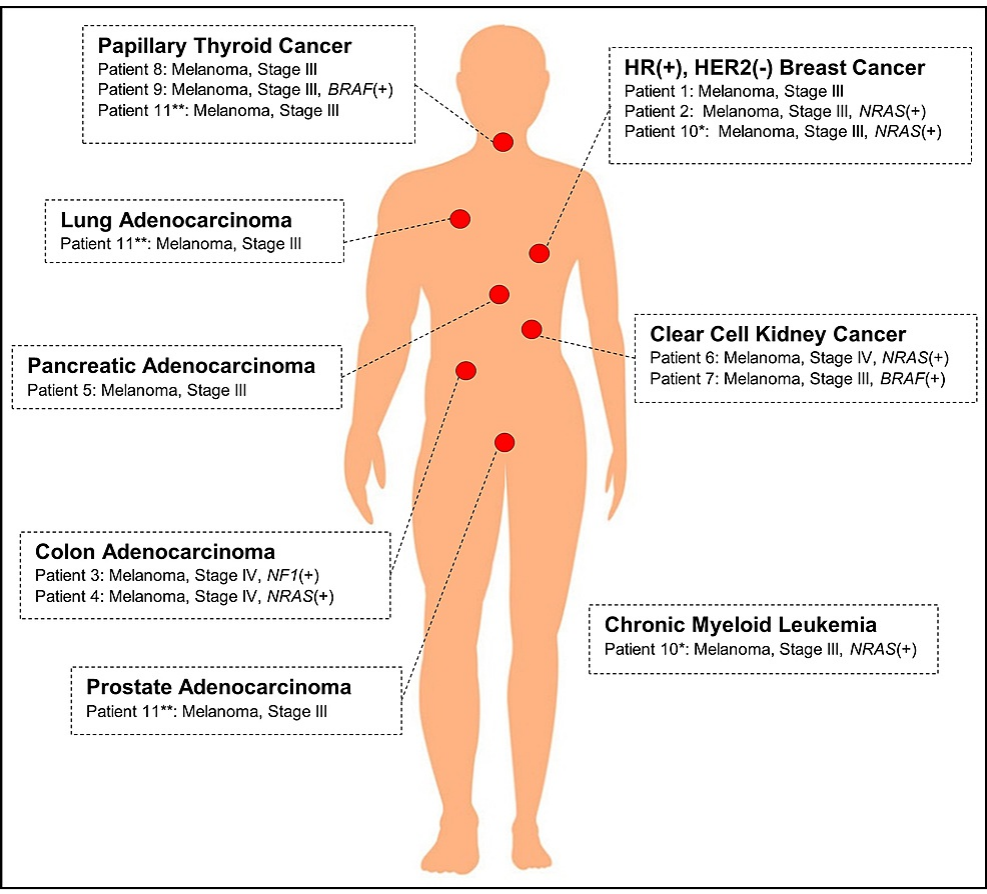
Distribution of non-melanoma primary malignancies and corresponding melanoma. Histopathology and somatic mutation status provided if available. BRAF – B-Raf proto-oncogene; HER2 – human epidermal growth factor receptor 2; HR – hormone receptor; NF1 – neurofibromin 1; NRAS – neuroblastoma Ras proto-oncogene. *Patient 10 had three primary malignancies. **Patient 11 had four primary malignancies.

The most common non-melanoma malignancies were breast and thyroid cancer (3 each); other cancers included colorectal (n = 2), kidney (n = 2), lung, chronic myeloid leukemia (CML), pancreas, and prostate (Figure 1). One patient had three primary cancers (Patient #10), while another patient had four (Patient #11). While three of four (75%) female subjects included in this study had breast cancer in addition to advanced melanoma, there was no specific cancer type that was most common amongst males. Second primary malignancies were localized in 45.5% of patients (n = 5), regionally advanced in 45.5% (n = 5), and metastatic in 27.3% (n = 3). Treatment of non-melanoma cancers included surgery (84.6%), radiation (15.4%), and systemic therapy (53.8%); systemic therapies comprised cytotoxics (57.1%), targeted therapy (28.6%), ICI (28.6%), or a combination (42.9%). Risk factors for MPM included viral infection (n = 1), tobacco (n = 1), and family history of malignancy (n = 6). Eight of 11 (72.7%) patients were referred for germline genetic testing; pathogenic variants were identified in three patients - BRCA1 (n = 1), BRCA2 (n = 1), and TYR (n = 1).

**Figure 2:**
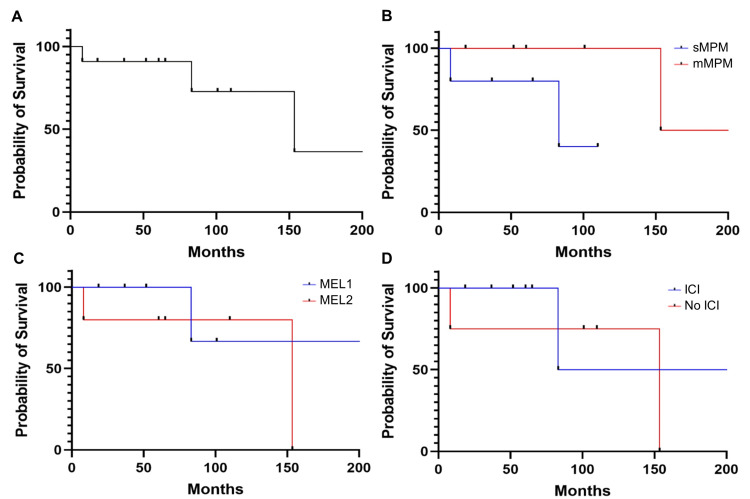
A) OS of patients with advanced melanoma and MPM. B) Comparison of OS between sMPM and mMPM. C) Comparison of OS between MEL1 and MEL2. D) Comparison of OS between patients treated with and without ICI for advanced melanoma.

The median OS for all patients was 153.5 months (Figure 2A). The median OS for sMPM and mMPM was 83.1 months and 196.7 months, respectively (p = 0.10) (Figure 2B). The median OS for MEL1 was not reached, in comparison to 153.5 months for MEL2; however, this was not statistically significant (p = 0.45) (Figure 2C). Among the 11 patients, 27.3% (n = 3) are deceased, while eight are alive. Notably six of these eight were treated with ICI for melanoma, with median OS 161.5 months, and 100% (6 of 6) achieving complete response with no evidence of melanoma. One patient (#10) who had three separate malignancies, including CML (in remission off tyrosine kinase inhibitor [TKI] therapy), and breast cancer is currently undergoing chemotherapy for breast cancer. There was no significant difference in median OS between patients receiving ICI and those who did not, however (p = 0.53) (Figure 2D).

## Discussion

MPM pose a complex challenge for the oncologist. It is well established that a) cancer survivors have higher risk of developing malignancy compared to the general population [7-10], and that b) second malignancies are an important cause of death for cancer survivors [10,11]. Furthermore, MPM account for roughly 5% of all cancers, and are increasing in incidence [12,13]. Yet MPM remain a poorly understood entity, not only in terms of clinical management, but also from a biological and mechanistic perspective.

Similar to other studies [3,14,15], we found that mMPM were more common than sMPM (54.5% vs 45.5%); we also noted a trend of improved survival for mMPM. In addition, MEL1 were more common than MEL2 (54.5% vs 45.5%); however, no significant difference in survival was observed. Breast and thyroid cancers were the most common non-melanoma cancers in our cohort. While limited by small sample size, we noted that 75% of female subjects included in this study had breast cancer as a second primary malignancy. Other studies have also reported lung, prostate and colon cancers as common MPM among patients with melanoma [16-18]. The mechanism for this distribution of MPM in association with melanoma is unclear. One possibility is simply that the most common cancers overall also occur as the most common second malignancies. However, a second possibility is that the most common second malignancies are those where underlying defects in host immunity and/or genetics may play a role. Melanoma is an immunogenic tumor where mechanisms of immune escape may include release of suppressive cytokines, down-regulation of surface antigens, lack of co-stimulatory function, and induction of tolerance [19]. Furthermore, there is evidence that immune suppression may facilitate metastatic progression of melanoma tumors [20]. One would expect that patients with defective antitumor immunity, and therefore already have a predisposition towards melanoma, are also predisposed to develop other, immunogenic tumors as well.

Since 2011, ICI have become the therapeutic mainstay for advanced melanoma [21-23]. Response rates to ICI approximate 40-65%, depending on whether single agent PD-1 or combined PD-1 and CTLA-4 inhibition is used, with complete responses of roughly 5-10%. Surprisingly, we observed exceptional responses among the six MPM patients treated with ICI; there was a complete response rate of 100% with all six patients now disease free from melanoma. Two of these six patients had a germline BRCA1 or BRCA2 pathogenic variant; recent data suggests that germline pathogenic variants affecting homologous recombination (HR) may modulate antitumor immunity [24]. We speculate that the underlying genetic predisposition to multiple malignancies may overlap with features that also lead to a robust anti-tumor immune response when treated with ICI. We note that mutations in HR genes occur in ~18% of melanoma patients [25], and thus may represent a unique group of patients to investigate in the context of ICI going forward.

While ICI are known to be efficacious against first primary tumors, their impact on second primary malignancies is not yet known. In a study by Heudel et al., patients treated with ICI for a first primary cancer had reduced risk of MPM, compared to those treated with chemotherapy [26]. In contrast, a SEER-based study comparing melanoma patients from the pre- and post-ICI eras (2005-2010 and 2011-2016, respectively), found that patients from the later period had more MPM [27]; however, the authors did not report whether patients received ICI or not (PD-1 inhibitors were approved in 2014), but rather looked at differences by date of treatment alone.

## Conclusions

This study highlights the fact that patients with advanced melanoma are at risk for multiple primary malignancies including breast and thyroid cancer. Furthermore, patients with secondary malignancies that occur as metachronous MPM may have better outcomes as opposed to synchronous MPM. And finally, given the role of the immune suppression in carcinogenesis, future studies are needed to study how the expanding use of ICI affects the occurrence of MPM going forward. We acknowledge the small sample size, retrospective nature of this study, and potential for lead time bias. Nevertheless, we emphasize that MPM patients are at high risk for poor outcomes and recommend further efforts to support this patient population, including genetic counseling to assess for underlying hereditary syndromes, careful history-taking for environmental exposure, and assessment for other causes of immune suppression. We also recommend a multi-disciplinary model, that includes a survivorship clinic that couples a personalized surveillance plan with risk-reduction strategies, such as sun protection, weight loss, tobacco cessation, age-appropriate cancer screening, and other wellness practices. Lastly, we propose that eligibility for clinical trial enrollment be broadened to include patients with mMPM, especially those who have demonstrated good outcomes following treatment of the initial malignancy.
